# Evolution and Conservation of *Bordetella* Intracellular Survival in Eukaryotic Host Cells

**DOI:** 10.3389/fmicb.2020.557819

**Published:** 2020-10-15

**Authors:** Israel Rivera, Bodo Linz, Eric T. Harvill

**Affiliations:** ^1^Department of Infectious Diseases, College of Veterinary Medicine, University of Georgia, Athens, GA, United States; ^2^Division of Microbiology, Department of Biology, Friedrich Alexander University Erlangen-Nuremberg, Erlangen, Germany

**Keywords:** *Bordetella*, evolution, intracellular survival, phagocytes, transcriptome

## Abstract

The classical bordetellae possess several partially characterized virulence mechanisms that are studied in the context of a complete extracellular life cycle in their mammalian hosts. Yet, classical bordetellae have repeatedly been reported within dendritic cells (DCs) and alveolar macrophages in clinical samples, and *in vitro* experiments convincingly demonstrate that the bacteria can survive intracellularly within mammalian phagocytic cells, an ability that appears to have descended from ancestral progenitor species that lived in the environment and acquired the mechanisms to resist unicellular phagocytic predators. Many pathogens, including *Mycobacterium tuberculosis*, *Salmonella enterica*, *Francisella tularensis*, and *Legionella pneumophila*, are known to parasitize and multiply inside eukaryotic host cells. This strategy provides protection, nutrients, and the ability to disseminate systemically. While some work has been dedicated at characterizing intracellular survival of *Bordetella pertussis*, there is limited understanding of how this strategy has evolved within the genus *Bordetella* and the contributions of this ability to bacterial pathogenicity, evasion of host immunity as well as within and between-host dissemination. Here, we explore the mechanisms that control the metabolic changes accompanying intracellular survival and how these have been acquired and conserved throughout the evolutionary history of the *Bordetella* genus and discuss the possible implications of this strategy in the persistence and reemergence of *B. pertussis* in recent years.

## Genus *Bordetella*

**Figure 1:**
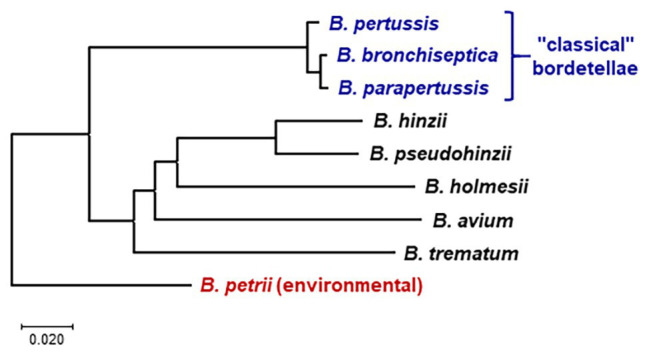
Whole genome phylogeny of nine *Bordetella* species based on pairwise average nucleotide identities (ANIs). The neighbor-joining tree was constructed from a distance matrix compiled from pairwise ANI between the genomes calculated at https://www.ezbiocloud.net/tools/ani (Yoon et al., 2017). The tree is drawn to scale in MEGA X (Kumar et al., 2018), with branch lengths in the same units as the evolutionary distances used to infer the phylogenetic tree. The tree was rooted according to the previously determined evolutionary relations between *Bordetella* species (Linz et al., 2016; Hamidou Soumana et al., 2017). *Bordetella petrii* is the only environmental among the analyzed species; all other species are human or animal pathogens.

The bordetellae are Gram-negative coccobacilli of the class Betaproteobacteria that are known to cause disease in a wide range of animals including small mammals and humans. Despite widespread vaccination, *Bordetella pertussis* – the etiological agent of whooping cough or pertussis – still affects tens of millions and causes hundreds of thousands of deaths, mostly in children younger than age 5, every year (Yeung et al., 2017). Characterized by paroxysmal cough accompanied by an inspirational whooping sound (hence the name), pertussis can last for months and cause severe respiratory complications and even death, particularly in infants and adults with underlying health conditions. *B. pertussis* is closely related to *Bordetella parapertussis* and *Bordetella bronchiseptica*, and the three species are collectively known as the “classical bordetellae” (Figure 1, Table 1). Multiple lineages appear to be host-restricted, with *B. pertussis* and *B. parapertussis* infecting only humans, although another independent lineage, named *B. parapertussis*_ov_ causes pneumonia in sheep (Porter et al., 1994; Park et al., 2012). In contrast, *B. bronchiseptica* appears to be more closely related to the progenitor of the classical bordetellae and is a respiratory pathogen of diverse mammals, causing a variety of pathologies ranging from chronic and often asymptomatic infection to acute bronchopneumonia and kennel cough in dogs (Goodnow, 1980).

**Table 1:** Genomic properties, host-specificity, and disease caused by *Bordetella* species.

Bordetella species	Host	Disease	Genome size (bp)	Intracellular survival
**Classical**				
*B. bronchiseptica*	Various mammals, including dogs, cats, pigs, rabbits, mice, horses, seals, sheep, and humans	Wide variety of respiratory disease, from clinically asymptomatic to acute pneumonia, such as kennel cough in dogs and rhinitis in pigs	5,338,400	Yes
*B. parapertussis*	Human-specific lineage; sheep-specific lineage	Whooping-cough-like disease in humans; pneumonia in sheep	4,773,551	Yes
*B. pertussis*	Humans	Whooping cough	4,086,551	Yes
**Non-classical**				
*B. avium*	Poultry and wild birds	Respiratory disease	3,732,255	No
*B. hinzii*	Poultry; immunocompromised humans	Respiratory disease (coryza) in poultry, septicemia in humans	4,885,897	Yes
*B. pseudohinzii*	Mice	Otitis media	4,490,371	Yes
*B. holmesii*	Humans	Whooping-cough-like infection, bacteremia	3,699,674	Unknown
*B. trematum*	Immunocompromised humans	Wound infection, skin disease	4,485,537	Yes
**Environmental**				
*B. petrii*	Environmental; immunocompromised humans	Environmental; wound and ear infection	5,287,950	Yes

In contrast to the closely related classical bordetellae, several *Bordetella* species with broader genetic diversity have been identified, collectively referred to as “non-classical” *Bordetella* (Figure 1, Table 1). Some members of the non-classical bordetellae are also known to be host specific animal pathogens. The emerging human pathogen *Bordetella holmesii*, initially isolated from the blood of septicemic patients (Weyant et al., 1995), has since increasingly been isolated from patients with pertussis-like respiratory infections (Yih et al., 1999; Rodgers et al., 2013). *Bordetella avium* causes respiratory infections in poultry and wild birds (Kersters et al., 1984). The other “avian” species, *Bordetella hinzii*, colonizes the respiratory tracts of poultry and was shown to cause disease during experimental infection in turkeys (Vandamme et al., 1995; Register and Kunkle, 2009), and has also been isolated from immunocompromised humans with respiratory disease and septicemia (Cookson et al., 1994; Funke et al., 1996). The closely related *Bordetella pseudohinzii*, identified as a pathobiont in mouse breeding colonies of commercial vendors (Ivanov et al., 2015, 2016), was found to cause chronic, transmissible otitis media in mice (Dewan et al., 2019). *Bordetella trematum*, an opportunistic human pathogen, can cause severe skin disease and chronic otitis media (Vandamme et al., 1996). The environmental species *Bordetella petrii*, originally isolated from an anaerobic bioreactor enriched with river sediment (von Wintzingerode et al., 2001), has subsequently been isolated from soil samples and also from immunocompromised patients (Fry et al., 2005; Nagata et al., 2015). These *Bordetella* species share many phenotypic characteristics that makes them successful animal pathogens.

Genome sequencing and multilocus sequence typing of the classical bordetellae revealed that *B. parapertussis* and *B. pertussis* independently evolved from a *B. bronchiseptica*-like ancestor (Parkhill et al., 2003; Diavatopoulos et al., 2005). Despite differences in host range and disease, the classical bordetellae are over 98% similar at the DNA sequence level and share many important virulence factors, including the well-known toxins such as adenylate cyclase toxin (ACT), pertussis toxin (PTX), and dermonecrotic toxin, and putative adhesins such as pertactin (PRN; Parkhill et al., 2003). Since these virulence factors are present in *B. pertussis*, *B. parapertussis*, and *B. bronchiseptica* but absent from the non-classical bordetellae, genes encoding these factors are believed to have been acquired before the divergence of the classical bordetellae (Park et al., 2012; Linz et al., 2016). Gain and loss of multiple genes, including those encoding bacterial toxins, protein secretion systems, and other virulence-associated factors, appear to have shaped the diversification and speciation in the genus. Gene loss has been more frequent than gene gain since their divergence, and loss of hundreds of genes was associated with the specialization of several host-restricted species, including the recently evolved human pathogens *B. pertussis*, *B. parapertussis*, and *B. holmesii* (Parkhill et al., 2003; Linz et al., 2016).

Gene loss and acquisition, *via* horizontal transfer, as a function of speciation and diversification is not restricted to the genus *Bordetella* and is well-documented in many other species and genera, including *Bordetella*’s sister genus *Achromobacter* (Li et al., 2013), other proteobacteria such as *Acinetobacter* (Chan et al., 2015), as well as bacteria from other phyla such as *Streptococcus* (Kilian and Tettelin, 2019) and *Mycobacterium* (Chiner-Oms et al., 2019). For example, several genome studies showed that horizontal transfer in the genus *Mycobacterium* has been a major player in shaping the species’ pathogenicity, leading to divergence (Ahmed et al., 2007; Saini et al., 2012; Panda et al., 2018). Thus, just like in other microbes, selective gene acquisition and loss has led to niche-specific adaptations that have shaped the metabolic versatility and pathogenicity in the genus *Bordetella*.

## Old Tricks New *Bordetella*

The genus *Bordetella* has largely been considered host-restricted pathogens with variable host-specificity. However, the recent discovery of several environmental species and 16S meta-analysis studies have revealed that the genus likely arose from an environmental origin (Hamidou Soumana et al., 2017). In addition, *B. bronchiseptica* was found to infect and persist within the amoeba *Dictyostelium discoideum*, to utilize its life cycle, translocating to the fruiting bodies and disseminating along with amoeba spores (Taylor-Mulneix et al., 2017a). These observations suggest that amoeba may represent an environmental niche for this, and possibly other animal-pathogenic *Bordetella* species and their progenitors. Similar to *B. bronchiseptica*, a wide variety of bacteria has been reported to form endosymbiotic relationships with amoebae, including bacterial genera as diverse as *Amoebophilus* (phylum *Bacteroidetes*), *Mycobacterium* (phylum *Actinobacteria*), *Parachlamydia* and *Protochlamydia* (phylum *Chlamydia*), *Caedibacter* (class *Alphaproteobacteria*), *Procabacter* (class *Betaproteobacteria*), and *Legionella* and *Caedibacter* (class *Gammaproteobacteria*; Molmeret et al., 2005; Schmitz-Esser et al., 2008; Samba-Louaka et al., 2018; Dubois et al., 2019). The interaction with amoebae as an environmental reservoir provides protection against external hazards, and possibly a competitive advantage against other bacteria, while enhancing bacterial dissemination along with the amoebic host. During interaction with amoeba, *B. bronchiseptica* undergoes phenotypic modulation inducing the expression of genes involved in chemotaxis, motility, and growth (e.g., flagella *flhD* and chemotaxis gene *cheZ*), while suppressing the expression of virulence factors such as the ACT (encoded by *cyaA*) and filamentous hemagglutinin (FHA) encoded by *fhaB* (Taylor-Mulneix et al., 2017a). Thus, in addition to possessing mechanisms to colonize a wide range of mammals including swine, rats, rabbits, sheep, dogs, and cats, *B. bronchiseptica* is able to establish a successful symbiotic relationship with amoeba in the environment outside a mammalian host.

The ability to adapt to profoundly different environments requires the capacity to sense and respond to changes in the surroundings. *Bordetella* species have evolved tools to rapidly modulate transcription in order to respond to such changes. Activation of virulence in *Bordetella* is largely controlled by the BvgAS two-component system, which consists of a sensor protein, BvgS, a transcriptional activator, BvgA, and a transcriptional repressor, BvgR. During growth at temperatures at and below 25°C, the sensor protein is unphosphorylated and inactive, and the bacteria are in the so-called Bvg minus (Bvg^−^) phase in which transcription of virulence genes is repressed. When receiving inducing signals such as temperature of 37°C, which mimics presence in a mammalian host, the BvgS sensor protein autophosphorylates, goes through a phosphorylation cascade and subsequently transfers a phosphor group to BvgA. Upon phosphorylation by BvgS, BvgA binds to the promoter regions of the Bvg-activated genes and induces the transcription of virulence genes in response to temperature, which results in the expression of virulence factors, such as the type six secretion system (T6SS), type three secretion system (T3SS), PRN, FHA, ACT, PTX, and others (Cummings et al., 2006; Nakamura et al., 2006; Nicholson et al., 2009; Hester et al., 2012).

## The Virulence-Repressed Bvg^−^ Phase And The Environment

Under laboratory conditions, the classical bordetellae, including *B. bronchiseptica*, can respond to different environmental stimuli by switching between two distinct lifestyles. When cultured at 37°C in the so-called Bvg^+^ phase, *in vitro* conditions that mimic the infectious phase at temperatures in the mammalian host (Luu et al., 2017), expression of genes associated with colonization and virulence, the so-called virulence activated genes, is upregulated (Moon et al., 2017; Chen and Stibitz, 2019). Activation of the Bvg^+^ phase is necessary and sufficient to facilitate bacterial colonization during infection. While in this phase, flagella and chemotaxis genes are repressed, and the bacteria are non-motile (Akerley et al., 1992). However, when cultured at 25°C, mimicking environmental conditions, *B. bronchiseptica* adopts a second lifestyle during which gene expression of virulence-related factors is repressed, while transcription of a large alternative set of genes is activated, the so-called Bvg^−^ phase. The biological significance of the Bvg^−^ phase has long been hypothetical, mainly due to the lack of a clear role for the virulence-repressed state during *in vivo* studies and lack of knowledge of *ex vivo* growth. Several authors have speculated that activation of virulence-repressed genes might serve a role during persistence in an environmental reservoir (Cotter and Miller, 1994; Hamidou Soumana et al., 2017; Moon et al., 2017; Taylor-Mulneix et al., 2017b; Linz et al., 2019). Since many of the Bvg^−^ phase transcribed genes are predicted to be metabolic enzymes and transport proteins, they are suspected to enhance acquisition of nutrients, growth, and proliferation in environmental settings. In addition, the transcriptionally active genes in the Bvg^−^ phase include chemotaxis and flagella synthesis genes, suggesting bacterial motility.

Indirect evidence and experimental data support the biological relevance of the Bvg^−^ phase in common ancestors of pathogenic *Bordetella*. First, in addition to the identification of numerous *Bordetella*-like bacteria among 16S ribosomal RNA (rRNA) sequences and metagenomes from soil samples (Wang et al., 2007; Hamidou Soumana et al., 2017; Garrido-Sanz et al., 2018), the animal-pathogenic *B. hinzii* and *B. bronchiseptica* were shown to grow efficiently in soil extract at 25°C (Hamidou Soumana et al., 2017). Second, using strains engineered to remain locked in a single phenotypic state by a point mutation in *bvgS* leading to constitutive phosphorylation (Bvg^+^ phase-locked mutant) or by *bvgS* gene deletion (Bvg^−^ phase-locked mutant; Cotter and Miller, 1994), the Bvg^−^ phase was shown to mediate *B. bronchiseptica* interactions with the soil amoeba *D. discoideum* (Taylor-Mulneix et al., 2017a). Tens of thousands of *B. bronchiseptica* wild-type and Bvg^−^ phase-locked bacteria were isolated from the amoeba fruiting bodies, in contrast to only 100 Bvg^+^ phase-locked *B. bronchiseptica*, indicating that the Bvg^−^ phase contributes to survival inside amoeba trophozoites and fruiting bodies (Taylor-Mulneix et al., 2017a). Interestingly, *B. bronchiseptica* not only survived but also multiplied inside amoeba fruiting bodies, as indicated by significantly increasing bacterial numbers over time, and were, then, disseminated along with amoeba spores by insects (Taylor-Mulneix et al., 2017a). The ability to switch between the Bvg^+^ and Bvg^−^ life styles appears to be conserved among the bordetellae (perhaps with the exception of *Bordetella ansorpii*), as *bvgA* and *bvgS* gene homologs have been identified in the genomes of animal-associated species as well as of the environmental *B. petrii* (Gerlach et al., 2004; Gross et al., 2008, 2010; Linz et al., 2016). Together, these observations suggest the presence of potential environmental reservoirs for many, if not most, animal-pathogenic and human-pathogenic *Bordetella* species. Although many *Bordetella* species are adapted to mammals, in which the Bvg^+^ phase is active, they still conserved the ability to respond to changes such as temperature fluctuations (Coote, 2001) by switching to the ancient Bvg^−^ phase for proliferation in environmental conditions.

Even though *B. pertussis* can phenotypically modulate between the two Bvg states (Stibitz et al., 1989; Moon et al., 2017; Chen and Stibitz, 2019), to this date, no evidence of an outside-host or environmental niche has been found for this human-restricted pathogen. This has led to the hypothesis that genes induced during the Bvg^−^ phase are likely the vestigial remnants of an important phenotype of ancient *Bordetella*. But although *B. pertussis* has lost over 20% of its genome since diverging from *B. bronchiseptica*, the Bvg regulon in *B. pertussis* is largely conserved, demonstrating that the system has been under purifying selection. In addition, the ability to switch between life styles seems to be conserved among the bordetellae, as *bvgA* and *bvgS* gene homologs have been found in the genomes of animal-associated species as well as of the environmental *B. petrii* (Gerlach et al., 2004; Gross et al., 2010; Linz et al., 2016, 2019), contradicting the “vestigial” hypothesis.

Recent work has shed some light onto the potential role of the Bvg^−^ phase in *B. pertussis*. First, Moon and colleagues analyzed *B. pertussis* gene expression under simulated Bvg^−^ conditions in the laboratory and identified the expression of genes involved in the fatty acid and lipid metabolism, of sugar and amino acid transporters, pyruvate dehydrogenase, genes of the phenylacetic acid catabolic pathway, and of the glycolate oxidation pathway (Moon et al., 2017). Moreover, peptidoglycan and lipopolysaccharide (LPS) synthesis genes as well as capsular polysaccharide biosynthesis loci were actively transcribed in the Bvg^−^ phase. Further, transcription of several genes encoding cold shock proteins was observed in this phase. These findings led the authors to speculate that transcriptional changes mediated by the switch from Bvg^+^ to Bvg^−^ in response to rapid temperature drops might be an important adaptation that facilitates survival outside the human host during transmission of *B. pertussis* (Moon et al., 2017). Second, as was shown in the baboon model of *B. pertussis* infection, transmission of *B. pertussis* occurs *via* airflow of aerosolized respiratory droplets (Warfel et al., 2012). Not surprising, this study also found that the distance between the infected host and the naive susceptible individual influences the rate of transmission. Thus, since *B. pertussis* must survive in airborne respiratory droplets to transmit to a new host, extended *B. pertussis* survival in cool air outside the host may be important for *B. pertussis* transmission (Trainor et al., 2015). And third, Bvg^−^ mutants were found to accumulate among a *B. pertussis* population in the nasopharynx of monkeys, suggesting an active role in facilitating bacterial persistence (Karataev et al., 2016). Taken together, these observations strongly suggest that the Bvg^−^ phase constitutes a conserved strategy that may have contributed to the survival of ancient *Bordetella*, and even though many *Bordetella* species have evolved to infect mammalian hosts, the ability to respond and adapt to environmental changes remains active. Thus, it appears likely that the virulence-repressed state indeed plays an active and important role in the biology of host-pathogenic *Bordetella* species.

## Intracellular Survival and Persistence

*In vitro* studies have shown that, upon entering human alveolar epithelial cell line A549, a significant portion of intracellular *B. pertussis* evades phagolysosomal fusion and remains viable in nonacidic compartments by a mechanism that is dependent on microtubule assembly, lipid raft integrity, and the activation of a tyrosine-kinase-mediated signaling (Lamberti et al., 2013). Numbers of viable intracellular bacteria increase from an average of one after 3 h to over five per A549 cell after 24 h. During this period, *B. pertussis* secretes a wide range of proteins involved in stress response, iron uptake, metabolism, and regulation, which allow the bacteria to reside and persist within host cells (Lamberti et al., 2013). Interestingly, intracellular survival appears to be dependent on the type of host cell, as viable *B. pertussis* were found to persist for 3 days in human macrophages and epithelial cells (Friedman et al., 1992; Lamberti et al., 2013) but less than 24 h in mouse DCs (Guzman et al., 1994a). *B. parapertussis* and *B. bronchiseptica* were also found to persist inside host phagocytes, emphasizing that the ability to survive intracellularly is not unique to *B. pertussis* (Guzman et al., 1994a; Gorgojo et al., 2012; Bendor et al., 2015).

**Figure 2:**
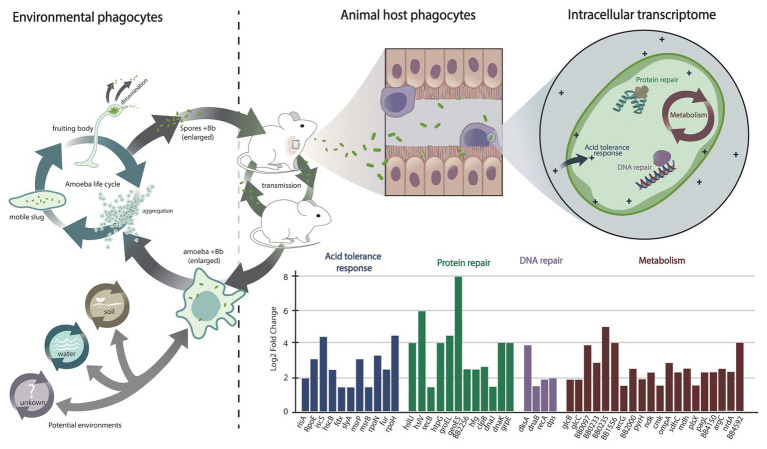
Interaction of *Bordetella* with environmental and animal host phagocytes and its transcriptional response during intracellular survival. Environmental phagocytes such as amoeba from soil, water, and other possible habitats (indicated by a question mark) represent an environmental reservoir for animal-pathogenic *Bordetella* species and their progenitors. *Bordetella* can resist digestion by amoebic phagocytes, translocate to the amoeba fruiting bodies, and disseminate along with amoeba spores. Animals and wind can spread bacteria and spores to new geographic locations, where the bacteria can either stay associated with the amoeba in a stable transmission cycle or infect a new mammalian host. Among the mammalian hosts, the bacteria can establish a transmission cycle that is independent but interconnected with the *Bordetella* life cycle in the amoebic host. During infection of a mammalian host, the bacteria are attacked by animal host immune cells, including macrophages. Uptake of *Bordetella bronchiseptica* by macrophages during infection of the mammalian respiratory tract triggers a bacterial SOS response that is marked by the expression of genes involved in protein repair, in DNA repair, and in acid tolerance. In addition, specific metabolic changes are associated with nutrient and oxygen deprivation, including transcriptional upregulation of genes of the glyoxylate cycle and downregulation of genes encoding proteins of the oxidative respiratory chain. In contrast to bacteria from other genera, the expression of genes involved in bacterial virulence is suppressed.

One aspect commonly overlooked is the role of the Bvg^−^ phase during intracellular persistence of *Bordetella* spp. inside host phagocytic cells. Spontaneous mutants lacking the *bvgS* gene, which encodes the sensor component of the BvgAS regulon, and the parental wildtype *B. bronchiseptica* displayed similar viability in DCs for over 72 h post infection. These data indicated that intracellular survival may be Bvg-independent or perhaps involves genes that are actively transcribed in the Bvg^−^ phase (Guzman et al., 1994a,b). Indeed, assessment of *B. bronchiseptica* gene expression during intracellular survival in macrophages supported these observations. Upon uptake by macrophages, *B. bronchiseptica* activates the expression of genes involved in protein repair, DNA repair, oxidative stress response, pH homeostasis, chaperone functions, and activation of specific metabolic pathways (Figure 2). By contrast, the expression of genes involved in bacterial virulence, which is a hallmark of Bvg-based modulation of gene expression in the mammalian host, is suppressed. None of the known virulence factors, including toxins, T6SS and T3SS, and the adhesins PRN and FHA, were transcriptionally active (Rivera et al., 2019), and neither were any of the 205 genes that were previously identified to encode proteins that are secreted under Bvg^+^ conditions (Luu et al., 2017). A similar expression pattern was recently reported for *B. pertussis* inside macrophages, where expression of genes encoding virulence factors such as the T3SS, ACT, PTX, PRN, fimbriae 2 (FIM2), tracheal colonization factor (TCF), and the serum resistance protein BrkA was also suppressed (Petrackova et al., 2020). Surprisingly, despite conducting these experiments at 37°C, a temperature known to induce the expression of virulence factors, both *B. bronchiseptica* and *B. pertussis* displayed strong suppression of genes associated with virulence. This seems to be a contradiction. However, all tested classical and non-classical *Bordetella* species with the sole exception of *B. avium* were able to persist in murine macrophages (Rivera et al., 2019); yet, the presence of virulence factors in *Bordetella* species was shown to be species-specific (Linz et al., 2016) due to multiple events of gene acquisition and gene loss in the genus. For example, the well-known virulence factors of the classical bordetellae, including PTX, are not present in the non-classical species (Linz et al., 2016).

In addition to BvgAS, other regulatory systems have also been implicated in promoting bacterial persistence inside macrophages. A two-component regulatory system named RisAS was found to be required for persistence of *B. bronchiseptica* inside spleen DC line CB1 and macrophage-like cell line J774A.1 (Jungnitz et al., 1998). Phenotypic exploration of a RisA mutant strain revealed that the RisAS regulon promotes resistance against oxidative stress, production of acid phosphatase, and *in vivo* persistence. RisA is optimally expressed at 37°C and when the bacteria are contained within eukaryotic phagocytes (Jungnitz et al., 1998). Notably, activation of RisA is regulated independently of BvgAS. Recent transcription analyses of RisA mutants under Bvg^+^and Bvg^−^ conditions showed that RisA very likely plays a major role during intracellular survival in mammalian phagocytes. The RisA-dependent *in vitro* transcription profiles (Coutte et al., 2016) were largely congruent with the transcriptional response of *B. bronchiseptica* and *B. pertussis* inside macrophages (Rivera et al., 2019; Petrackova et al., 2020). Under Bvg^+^ conditions, BvgA is phosphorylated and induces transcription of virulence-activated genes as well as of the transcriptional repressor BvgR. BvgR has diguanylate phosphodiesterase activity, which degrades intracellular c-di-GMP to GMP. In the Bvg^−^ phase, and in the absence or at very low levels of c-di-GMP, unphosphorylated RisA activates transcription of a variety of genes, including those involved in osmotic and oxidative stress response, pH homeostasis, and chaperone functions such as *groEL* (Coutte et al., 2016). At the same time, expression of many iron-regulated genes including *tonB* is suppressed. At sufficiently high levels of intracellular c-di-GMP, RisA binds to c-di-GMP. Unphosphorylated, c-di-GMP-associated RisA also suppresses expression of iron genes. In contrast, phosphorylated c-di-GMP-binding RisA initiates expression of many virulence-repressed genes, including those for O-antigen and capsule biosynthesis. In addition, phosphorylated and c-di-GMP-bound RisA not only inhibits the expression of the virulence-activated genes but also suppresses the expression of flagellar and chemotaxis genes (Coutte et al., 2016). Thus, the interplay between BvgA and RisA emphasizes the complexity of the regulatory network required for bacterial adaptation to the host intracellular environment.

In addition, the avirulent Bvg^−^ phase has been shown to be required for survival, persistence, and replication of *B. bronchiseptica* within amoeba (Taylor-Mulneix et al., 2017a). It is plausible that while many pathogenic bacteria respond to stress by inducing the expression of virulence, *Bordetella* species employ an ancient conserved stress response to cope with the challenges of an intracellular environment. These observations suggest a critical role of the Bvg^−^ phase in modulating gene expression during bacterial interaction within host phagocytes. However, despite these findings, it is important to recognize that these observations only constitute a snapshot of a rather dynamical and complex process. The above studies at the messenger RNA (mRNA) level have shown suppression of virulence upon internalization in macrophages. In contrast, similar analyses of the *B. pertussis* proteome reported unchanged abundance of virulence proteins PTX and ACT in intracellular *B. pertussis* compared to *in vitro* grown under Bvg^+^ conditions (Lamberti et al., 2016; Valdez et al., 2016), which was interpreted as these proteins contributing to bacterial persistence in macrophages. This apparent contradiction may be due to slow degradation of the already synthesized proteins, which would suggest that the protein levels might decrease at later time points. In this case, the avirulent Bvg^−^ phase would represent the authentic phenotype during intracellular survival.

The ability to survive inside macrophage spans beyond the classical bordetellae (Rivera et al., 2019). Comparative genome analyses between *B. bronchiseptica* and non-classical *Bordetella* species revealed the conservation of genes involved in intracellular persistence; approximately 80% of the 318 transcriptionally upregulated *B. bronchiseptica* genes during intracellular persistence were present in the genomes of non-classical *Bordetella* spp., in contrast to ~50% of the total of 4,981 evaluated *B. bronchiseptica* genes. Phenotypic analyses validated the significance of this observation by demonstrating that both the classical and the non-classical *Bordetella* spp. can persist inside RAW 264.7 macrophages (Rivera et al., 2019). The shared ability for intracellular persistence, and evolutionary conservation of the genes suspected to be involved in it, strongly suggests that intracellular survival represents an ancestral trait, the origin of which precedes speciation in the genus. As such, this feature must have evolved at some point during evolution of *Bordetella* spp. from environmental bacteria to mammalian respiratory pathogens. *B. bronchiseptica* and also the sheep-associated *B. parapertussis*_ov_ can resist digestion and utilize the life cycle of the ubiquitous soil amoeba *D. discoideum* to multiply and disseminate (Taylor-Mulneix et al., 2017a,b). The ancient interaction with these and potentially other environmental phagocytes could have been an important evolutionary milestone on the way to intracellular survival in phagocytic cells. Thus, intracellular persistence in environmental phagocytes may have been a “training ground” – as some authors named it – for the subsequent evolution of bacteria, including *Bordetella* spp., to animal pathogens (Greub and Raoult, 2004; Molmeret et al., 2005; Salah et al., 2009; Taylor-Mulneix et al., 2017b). While this point of view of an ancient evolutionary adaptation preceding speciation suggests that many, if not all, animal-pathogenic *Bordetella* species can interact with amoeba, this prediction remains to be evaluated.

## Metabolic Changes During Intracellular Survival

Internalization by macrophages triggered a strong general stress response known as SOS response (Simmons et al., 2008) in *B. bronchiseptica* (Figure 2), characterized by suppression of cell division *via* downregulation of the *fts* locus and by upregulation of DNA repair genes, of protein chaperone genes, of oxidative stress response, and of acid tolerance genes (Rivera et al., 2019). As expected under microaerophilic/hypoxic conditions inside macrophages, transcription of the *nuo* genes that encode the oxidative respiratory chain was strongly suppressed. In contrast, genes of the glyoxylate cycle displayed elevated expression, including the gene *glcB* encoding malate synthase G and its transcriptional activator *glcC*, malate dehydrogenase gene *mdh*, citrate synthase gene *gltA*, and aconitase gene *acnB* (Rivera et al., 2019). The glyoxylate cycle is important in the utilization of acetate or fatty acids as the main carbon source and may be essential for nucleotide and amino acid biosynthesis under intracellular conditions (Munoz-Elias and McKinney, 2006). The avian pathogen *B. avium* is the only animal-adapted *Bordetella* species that lacks malate synthase transcriptional regulator *glcC*, and this species was severely impaired in its ability to persist in macrophages. Indeed, deletion of this gene in *B. bronchiseptica* resulted in a significant reduction in intracellular survival, but complementation of the knockout mutant with a plasmid-borne gene copy restored the wild-type phenotype, confirming a critical role of *glcC* during intracellular persistence (Rivera et al., 2019). Besides the glyoxylate cycle, several genes of fatty acid synthesis pathways such as 3-oxoacyl-ACP reductase BB4150, long chain fatty acid Co-A ligase BB0233, outer membrane protein OmpA, and ABC transport protein encoded by BB1556 were found to be strongly induced, suggesting that increased membrane biosynthesis. In addition, elevated expression of genes involved in amino acid biosynthesis and transport, of *de novo* nucleotide biosynthesis as well as of numerous ribosomal protein genes were indicative of general extensive metabolic activity in the bacterial cell in response to internalization by macrophages (Rivera et al., 2019).

Notably, the array of genes expressed during intracellular survival holds similarity to those expressed in response to low pH exposure (pH less than 4) following acid adaptation (pH at 5.5; Fingermann and Hozbor, 2015). Synthesis of ribosomal proteins (ribosomal protein L1, L4, and L5), molecular chaperones (GroEL, HSP 90, and DnaK), proteases (BB3293 and BB1248), metabolic related proteins (succinate-semialdehyde dehydrogenase, aconitate hydratase, and argininesuccinate synthase), and LPS modification have been reported to contribute to *B. bronchiseptica* acid tolerance response. Furthermore, *B. bronchiseptica* grown in the avirulent phase or a Bvg^−^ phase-locked strain displayed greater resistance to lethal acid challenge, and thus, increased survival rate in acid. These similarities suggest that upon entry in host macrophages, *B. bronchiseptica* modulates the transcription of genes involved in the acid tolerance response while suppressing the expression of virulence genes as a mechanism to enhance resistance against stringent acid conditions.

Interestingly, the activation of an acid tolerance response is one of the many features utilized by pathogens known to cause human infection and persist inside host phagocytes, including macrophages and DCs (Thakur et al., 2019). For example, *S. enterica* serovar Typhimurium can subvert macrophage mediated killing and survive within the acidic environment of macrophages by altering lysosomal pH. Several regulatory proteins involved in reduction of acidic pH and activation of the stress response have been previously identified for intracellular *S. enterica*. These includes the environmental response regulator protein sigma factor RpoH, OmpR, and ferric uptake regulator protein (Fur), and stress response proteins alkyl hydroperoxide reductase (AhpC), DNA protection during starvation protein (Dps), recombinase A (RecA), and Mg(2+) transport ATPase protein C (MgtC; Imre et al., 2013; Lund et al., 2014). Expression of gene homologs encoding for RpoH, OmpR, Fur, AhpC, Dps, and RecA has been previously identified for intracellular *B. bronchiseptica* (Rivera et al., 2019), while MgtC has been reported to contribute to *B. pertussis* persistence inside macrophages (Lamberti et al., 2016; Cafiero et al., 2018).

In many bacteria including *Brucella* spp., *Listeria monocytogenes*, *S. enterica*, *Vibrio cholerae*, as well as *B. pertussis* and *B. bronchiseptica*, another important stress response protein is the RNA-chaperone protein Hfq (Robertson and Roop, 1999; Christiansen et al., 2004; Ding et al., 2004; Sittka et al., 2007; Bibova et al., 2013, 2015; Lamberti et al., 2016; Rivera et al., 2019; Alvarez Hayes et al., 2020). Hfq regulates the binding of non-coding RNAs to mRNAs, which can result either in translational repression and mRNA degradation or in activation of translation. Hfq was found to mediate the response to osmotic and heat stress, as well as to starvation in the stationary growth phase (Robertson and Roop, 1999). Besides stress resistance, Hfq plays a role in the post-transcriptional regulation of virulence factor expression (Christiansen et al., 2004; Sittka et al., 2007). For example, in *B. pertussis*, an Hfq deletion mutant displayed reduced expression of the TCF and the T3SS (Bibova et al., 2015). In addition, Hfq deletion mutants were found to be severely impaired in their ability to survive inside macrophages (Bibova et al., 2013; Alvarez Hayes et al., 2020), suggesting an important role of the Hfq-mediated stress response during intracellular survival.

Similar to *B. bronchiseptica*, *Francisella tularensis* – a facultative intracellular pathogen and the causative agent of tularemia – can infect and proliferate within mammalian phagocytes as well as in amoeba (Abd et al., 2003; El-Etr et al., 2009; Ozanic et al., 2015). Upon infection, intracellular *F. tularensis* subverts host defenses by escaping the phagosome (Celli and Zahrt, 2013) or by synthesizing proteins that promote persistence inside macrophages, such as macrophage growth locus protein MglA, stringent starvation protein A (SspA), chaperone HtpG, carbamoyl-phosphate synthase (CarA), and iron binding rubredoxin (RubA; Fuller et al., 2008; Charity et al., 2009; Bell et al., 2010; Chong and Celli, 2010; Dai et al., 2010; Bent et al., 2013). Previous work has demonstrated that intracellular *B. pertussis* can avoid the phagosome-lysosome fusion and replicate inside macrophages within 48 h post infection (Lamberti et al., 2010). In addition, upregulation of genes encoding for MglA (1.7 fold-change), AroG (2.7 fold-change), HtpG (4.2 fold-change), CarA (1.5 fold change), RubA (2.1), and to a lesser degree for SspA (1.2 fold change) has been reported for intracellular *B. bronchiseptica* at 2 h post infection of RAW 246.7 macrophages (Rivera et al., 2019).

For intracellular pathogens, the ability to persist inside host-phagocytes is contingent upon adjustment to the intracellular environment. Lethal pH, resource starvation, and oxidative burst are among the threats intracellular bacteria will face upon entry in host cells, which often result in damage to protein and DNA integrity and nutrient depravation. Mechanisms commonly employed by intracellular bacteria such as *L. monocytogenes*, *S. enterica*, and *Clostridium* spp. include the synthesis of protein complexes that promote bacterial cell homeostasis under demanding environmental conditions (Lund et al., 2014). Synthesis of molecules such as recombinase RecA, chaperones DnaK, GroEL, GroES, Hsp90, and protease Clp promotes DNA repair and folding of critical enzymes and removal of damage proteins, thus maintains cell integrity upon stress. Like in many intracellular facultative pathogens, the presence of genes encoding for these proteins in the *Bordetella* genome, and their elevated expression during survival inside macrophages, serves as evidence that the bacteria possess the mechanisms to cope with the hazardous condition often present inside host cells, including professional phagocytic cells (Rivera et al., 2019).

## Impact Of Intracellular Survival During Infection *In Vivo*

Host response to *Bordetella* spp. infections has largely been studied in mice challenged with *B. pertussis* or with its close relative *B. bronchiseptica*. Upon infection, *B. pertussis* secretes a wide array of virulence factors that among other functions, mediate adherence to host epithelial cells and promote bacterial survival in the host respiratory track.

For instance, *B. pertussis* internalized by host cells can persist intracellularly within ciliated epithelial cells and alveolar macrophages. Studies in murine models have shown that innate immune cells and antimicrobial peptides help to control the infection, while complete bacterial clearance requires cellular immunity mediated by T-helper type 1 (Th1) and Th17 cells (Mills et al., 1999). During the early stages of infection, local and innate immune cells including macrophages, DCs, neutrophils, and natural killer (NK) cells are recruited and largely accountable for the control and reduction of *B. pertussis* (Carbonetti, 2007; Carbonetti et al., 2007; Higgs et al., 2012). Among the effectors that promote bacterial reduction, early secretion of interferon-gamma (INF-γ) by NK cells, DC, and Th1 cells greatly enhance macrophage-mediated killing of *B. pertussis*; mice lacking INF-γ developed a lethal infection after challenge with *B. pertussis* (Mahon and Mills, 1999; Higgins et al., 2006).

The adaptive immune system plays a crucial role in clearance of *B. pertussis* infection. Activation and recruitment of a Th1 and Th17 adaptive immune response mediates bacterial clearance from the lower and upper respiratory track of mice. Modulation of cellular Th1 immunity results in the secretion of IL-12 and INF-γ, which in turn promotes neutrophil recruitment and enhances macrophage phagocytic activity. It is largely recognized that optimal immunity to primary and secondary *B. pertussis* infections is conferred by the priming and activation of Th1 cells. Similar to convalescent immunity, vaccination with whole cell pertussis (wP) – which is based on heat-killed *B. pertussis* bacteria – promotes the secretion of Th1 derived cytokines and enhances macrophage activity, resulting in increased killing of phagocytosed bacteria. Protection conferred by wP has been widely studied, and the rapid decline in the number of pertussis cases following its introduction is strong and compelling evidence for its effectiveness. However, while whole cell pertussis vaccines (wP) were highly effective at clearing the infection and providing long-term protection, several cases of vaccine reactogenicity were reported, which led to its replacement in many countries by acellular vaccines (aP) that are composed of three to five purified immunogenic antigens. Even though acellular pertussis vaccine is effective at clearing *B. pertussis* infection from the lungs and protecting against disease pathology, vaccination with aP, in contrast to immunization with wP vaccine, does not provide protection against bacterial colonization of the upper respiratory tract of mice and fails to prevent transmission among non-human primates (Warfel and Merkel, 2013; Warfel et al., 2014).

Despite wide vaccination coverage, the incidence of pertussis is increasing, which prompted the National Institute of Allergy and Infectious Diseases (NIAID) to add *B. pertussis* to the list of priority emerging infectious diseases/pathogens in 2015. Multiple factors have been suspected to contribute to the reemergence of pertussis. These include greater awareness and reporting, improved detection methodology, vaccine hesitancy and incomplete vaccine boosters in some areas, antigenic shifts in *B. pertussis* to escape vaccine-mediated immunity, more rapid waning of aP-induced immunity and decreased long-term protection compared with whole cell vaccines, and a less effective aP-induced immune response that does not prevent colonization and transmission of *B. pertussis*, resulting in increased carriage and asymptomatic transmission (Cherry, 2012; Klein et al., 2012; Bart et al., 2014; Warfel et al., 2014; Althouse and Scarpino, 2015; Dewan et al., 2020). As outlined above, infection with *B. pertussis* naturally induces a significant Th1-type T-lymphocyte cytokine response in mice that is characterized by high levels of IL-2, IFN-γ, and TNF-α, a type of immune response that is characteristic of infection by intracellular pathogens (Spellberg and Edwards, 2001; Thakur et al., 2019). While protective immunity generated by wP vaccination also promotes a Th1 immune response, the less efficacious aP vaccines induce a strong Th2 and antibody response. Since the aP vaccine-induced Th2 response does not effectively target intracellular pathogens, we propose that the inability to clear intracellular bacteria could allow *B. pertussis* to evade host immunity during survival inside phagocytic cells. This would protect the bacteria from antibodies, complement activation, and bactericidal activity, and thus, would result in suboptimal vaccine protection. Thus, given that the number of pertussis cases have been on the rise following the widespread use of aP vaccines, it seems plausible that an inadequate immune response to intracellular *B. pertussis* may have been contributing to the reemergence of whooping cough.

Whooping cough is caused by *B. pertussis* and, to a lesser extent, *B. parapertussis*. Similar to *B. pertussis*, the incidence of whooping cough-like disease caused by *B. parapertussis* has been increasing over the past decades. Antibodies elicited against the wP vaccine were shown to also target *B. parapertussis*, and thus, provide some level of protection, even though less protection than against *B. pertussis* (Willems et al., 1998; Zhang et al., 2009). In contrast to wP vaccines, aP-elicited antibodies showed no efficacy against *B. parapertussis*. On the contrary, aP vaccination induced host immunity that somehow interfered with the optimal clearance of *B. parapertussis*. Therefore, it was speculated that widespread aP vaccination against *B. pertussis* might increase the risk of acquiring an *B. parapertussis* infection (Long et al., 2010). Further studies revealed that the *B. parapertussis* O-antigen inhibited antibody binding to the bacteria (Zhang et al., 2009; Gorgojo et al., 2012). In the absence of opsonizing antibodies, neutrophils were unable to kill *B. parapertussis* after phagocytic uptake. A high proportion of the engulfed bacteria persisted in nonacidic phagosomes, establishing an intracellular infection (Gorgojo et al., 2012, 2014). Intracellular survival and persistence of *B. parapertussis* is tempting us to speculate that the inability of the aP vaccine-induced Th2 response to effectively target intracellular pathogens may be among the reasons for the increasing incidence of *B. parapertussis*.

Pathogenic members of the genus *Bordetella* have adapted to colonize, replicate, and transmit in animal hosts. The environmental origin of the species suggests that the ability to survive and persist in environmental phagocytes could have protected the bacteria from external dangers while ensuring transmission to novel environments and hosts. The interaction of the bacteria with environmental amoeba may have forced the evolution of schemes that allowed them to interact with mammalian phagocytes, specifically the evolution of tools to avoid and survive phagocytic digestion. Thus, a once critical survival strategy in the environment could potentially affect the success of the bacteria during mammalian host infection. This mechanism to evade the host immune system appears to be contributing to their emergence as important animal and human pathogens. Interestingly, similar to the interaction of *B. bronchiseptica* with amoebae (Taylor-Mulneix et al., 2017a), the transcriptional response to internalization by mammalian phagocytes occurred in the environmental and avirulent Bvg^−^ phase (Rivera et al., 2019; Petrackova et al., 2020). Despite the interaction with macrophages at 37°C, the mammalian host temperature, and thus, supposedly Bvg^+^ conditions, transcription of the well-known virulence factors was downregulated. While this seems contradictory, it makes sense because the presence of virulence factors in *Bordetella* species is largely species-specific (Linz et al., 2016) but both classical and non-classical *Bordetella* species were able to persist in murine macrophages (Rivera et al., 2019). Evolution and host specialization, such as the evolution of *B. pertussis* from a *B. bronchiseptica*-like ancestor and its adaptation to a closed transmission cycle with only a human host (Dewan and Harvill, 2019), were often accompanied by genome reduction. In case of *B. pertussis* and the human lineage of *B. parapertussis*, this loss of hundreds of genes ended their ability to utilize amoebae as an environmental reservoir (Taylor-Mulneix et al., 2017b). By contrast, *B. pertussis*’ and *B. parapertussis*’ ability to persist in macrophages was preserved, likely because of strong selection for a favorable trait in the mammalian/human host. Careful examination of the mechanisms by which *Bordetella* reside inside host cells and the role of intracellular persistence during infection can provide valuable insight and new approaches to confront the reemergence of whooping cough.

## Author Contributions

IR, BL, and EH wrote the review. IR and BL contributed equally to this manuscript and should be considered equally contributing first authors. All authors approved the submitted version.

### Conflict of Interest

The authors declare that the research was conducted in the absence of any commercial or financial relationships that could be construed as a potential conflict of interest.

The reviewer MR declared a past co-authorship with the author EH to the handling editor.
